# Bell Palsy Mimics: Lessons from Four Malpractice Cases

**DOI:** 10.5811/cpcem.38451

**Published:** 2025-04-05

**Authors:** Rachel Lindor, Summer Ghaith

**Affiliations:** * Mayo Clinic, Department of Emergency Medicine, Rochester, Minnesota; † Mayo Clinic, Mayo Clinic Alix School of Medicine, Phoenix, Arizona

**Keywords:** Bell palsy, malpractice, medicolegal, lawsuit

## Abstract

**Introduction:**

Bell palsy, an idiopathic dysfunction of the seventh cranial nerve, is the leading cause of unilateral facial paralysis, although other more serious entities such as stroke, infection, and tumor may present similarly, leading to both medical and legal risks in cases of misdiagnosis.

**Case Series:**

We present four malpractice cases revolving around misdiagnosis of Bell palsy. These cases alleged failure to diagnose, failure to obtain informed consent, and failure to provide appropriate discharge instructions. Outcomes ranged from a jury verdict in favor of the physician, to an out-of-court settlment for $400,000, to a jury verdict in favor of the patieint for over $3.1 million.

**Conclusion:**

Bell palsy is the most common cause of unilateral facial paralysis. While the diagnosis can be made at the bedside without advanced testing, doing so requires a clear understanding of the pathophysiology of the disease, an appreciation for the role of advanced diagnostics, and thorough documentation of a supportive history and physical exam. Misdiagnosis or mismanagement confers both clinical and legal risks.

## INTRODUCTION

Bell palsy, often diagnosed in the emergency department (ED), is a relatively benign, idiopathic condition that manifests as unilateral facial weakness, thought to be secondary to inflammation of the seventh cranial (facial) nerve.^1^,^2^ It is the most common cause of unilateral facial paralysis, a symptom also seen in other serious conditions such as stroke, infections, and tumors.^1^ The diagnosis generally can be made based on history and physical examination, which often demonstrates fairly abrupt onset of unilateral symptoms involving weakness or paralysis of facial muscles including the forehead, a sensation of facial numbness, alterations in taste, and hyperacusis. Differential diagnoses include any other conditions that may affect the cranial nerve including stroke, tumors, vasculitides, and infections such as Lyme disease and herpes viruses.^3^

Given the broad spectrum of causes of facial paralysis, the opportunities to misdiagnose Bell palsy are numerous. The literature suggests the misdiagnosis rate to be anywhere from 1%–20%.^3^,^4^,^5^ Given the multiple dangerous and treatable etiologies that may mimic Bell palsy, the clinical and legal risks of misdiagnosis are high. Here, we discuss four medical malpractice cases centered around Bell palsy, drawing attention to common clinical characteristics of missed diagnoses and steps clinicians can take that may mitigate their malpractice risks.

## CASE SERIES

### Case 1: *Hamilton*

A 60-year-old male presented to the ED with slurred speech, left-sided facial weakness, and deviation of his tongue. The defendant emergency physician diagnosed the patient with Bell palsy. After discharge, the patient’s symptoms worsened, and he was subsequently diagnosed with a stroke. He alleged that the physician’s failure to diagnose this at his first visit led to his poor outcome, including permanent motor and cognitive impairment. The case settled for $400,000.^6^

### Case 2: *Taite*

A 42-year-old male with a history of hypertension and diabetes presented to the ED with right-sided facial droop, slurred speech, and dizziness. He was initially examined by a resident physician, who diagnosed him with Bell palsy based on his lack of arm or leg weakness. No imaging was obtained, a supervising physician agreed with the assessment, and the patient was discharged. The next day he returned with more severe symptoms and was found to have had a large hemorrhagic stroke, from which he subsequently died five days later. After two years of litigation and a jury trial, the physicians in this case were ultimately found not negligent in the care provided.^7^

### Case 3: *Jandre*

A 53-year-old male presented to the ED with dizziness, slurred speech, and facial weakness. The defendant emergency physician diagnosed the patient with Bell palsy and discharged the patient. The patient developed permanent neurologic deficits secondary to a stroke and sued the physician both for failing to diagnose and treat the stroke and for failing to inform the patient of additional options for diagnosis. The case went to trial, and a verdict was rendered for $3,106,433.^8^

### Case 4: *Neu*

A 59-year-old female was diagnosed with Bell palsy after presenting for evaluation of ear pain and facial weakness. No imaging was performed. No follow-up was recommended. The patient’s symptoms persisted, and she was ultimately diagnosed with parotid gland cancer eight months later, which had widely metastasized by that time. The lawsuit, alleging failure to obtain appropriate imaging and failure to diagnose, was ultimately unsuccessful as it was filed after the statute of limitations.^9^

## DISCUSSION

We present four cases involving serious conditions that were misdiagnosed as Bell palsy. While Bell palsy is generally considered a clinical diagnosis, meaning a condition that can be identified based on a clinical history and physical exam, being able to make this diagnosis confidently requires thoroughly understanding the pathophysiology of the condition and recognizing when a bedside exam is not sufficient. As stated above, Bell palsy is thought to be due to inflammation of the seventh cranial nerve, which normally controls a) muscles of the face (raising eyebrows, shutting eyelid, wrinkling nose, smiling); b) muscles of the middle ear that regulate sound; c) taste on the anterior two-thirds of the tongue; d) output from the submandibular and sublingual salivary glands; and e) output from the lacrimal ducts.

Traditionally, Bell palsy is diagnosed in patients with dysfunction of the seventh cranial nerve who may present with unilateral facial weakness or paralysis, hyperacusis, change in normal taste perception or saliva production, and change in tear production. Some patients endorse pain near the ear on the affected side overlying the facial nerve itself. These symptoms tend to appear rather abruptly and reach maximum intensity within hours to days. Symptoms that fall outside this constellation should prompt clinicians to consider alternative diagnoses. Treatment consists of corticosteroids to address the inflammation with or without concomitant antiviral therapy.^10^ The majority of patients will recover full function, while about 5–20% will have persistent deficits.^3^ Those who do not show response to treatment within three weeks warrant additional evaluation to assess for alternative causes.^11^ Treatable diagnoses that may present with overlapping symptoms include ischemic stroke, hemorrhagic stroke, intracranial tumor, extracranial tumor, viral infections (eg, herpes), and bacterial infections (eg, Lyme disease). Being aware of these mimics is critical in not overlooking their presence.

**CPC-EM Capsule**

What do we already know about this clinical entity?

*Bell Palsy, often diagnosed in the emergency department (ED), causes unilateral facial weakness due to facial nerve inflammation but can mimic serious conditions like stroke or tumors*.

What makes this presentation of disease reportable?

*Four malpractice cases on Bell Palsy highlight characteristics of missed diagnoses and key steps clinicians can take to reduce liability*.

What is the major learning point?

*Clinicians should identify atypical Bell Palsy symptoms, like tongue deviation and dizziness, and recognize risk factors for conditions like acute stroke*.

How might this improve emergency medicine practice?

*Careful history, exam, and documentation, including shared decision-making and discharge instructions, can aid accurate diagnosis and reduce legal risks*.

Clinicians should be adept at recognizing patients who have symptoms that are not consistent with Bell palsy. For example, in the first case, the patient presented with tongue deviation. The muscles of the tongue are controlled by the hypoglossal nerve, cranial nerve XII, not the facial nerve, as is affected in Bell palsy. Identification of tongue deviation in the setting of a unilateral facial paralysis suggests a central process affecting multiple cranial nerves and warrants a more thorough evaluation. Maintaining a solid knowledge of the facial nerve anatomy and function is crucial in recognizing these subtle differences, and meticulously documenting this exam is critical for supporting a bedside diagnosis.

In the second and third cases, a patient presented with unilateral facial weakness and slurred speech, symptoms that could be consistent with Bell palsy, but they also endorsed dizziness. Dizziness is the most overlooked symptom in missed diagnosis of strokes and is not consistent with the diagnosis of Bell palsy.^12^ Any co-occurrence of dizziness should prompt evaluation for alternative diagnoses. A review of 69 malpractice cases of reported dizziness as a missed symptom of acute stroke revealed that patients had poor outcomes due to a missed or delayed central nervous system diagnosis, most commonly in the ED setting.^13^ The majority of these patients exhibited at least one additional neurologic symptom in addition to dizziness, as did the patient in this malpractice case.^13^ These findings underscore the importance for emergency physicians to consider alternative explanations in patients presenting with dizziness or any other symptoms that do not fit into the constellation of symptoms caused by dysfunction of the facial nerve.

The patient in the second case also had multiple risk factors for stroke, including hypertension, diabetes, and polysubstance use. One study analyzing ED misdiagnoses of Bell palsy found that more than one-quarter of these misdiagnoses were ischemic stroke.^5^ While the diagnosis of Bell palsy can usually be made without advanced diagnostics, all patients—especially those with stroke risk factors—require careful consideration and documentation. In this case, we are told that physicians relied on the patient’s lack of arm or leg weakness to exclude a stroke. This is not a sufficient clinical history or exam in any patient, but especially not in a patient with a higher pre-test probability for stroke based on his risk factors. History and exam focused on ruling out more dangerous etiologies *and* thorough documentation of both of these is crucial.

In the third case, from Wisconsin, the court found the physician to be negligent in part for not discussing the option of advanced imaging with the patient whose symptoms were atypical for Bell palsy. That court determined that the physician should have engaged in an informed consent discussion with the patient, stating that the physician has a duty to disclose diagnostic options that reasonable patients would want to know to make informed decisions about their care.^8^ This finding is remarkable in that it seemingly extends the scope of traditional informed consent beyond the historical focus on treatments and also applies it to diagnostic tests. While this court decision applies only in Wisconsin, it may be indicative of a larger shift in the courts toward a more patient-centered standard of care in which patients are expected to be invited to be a part of the decision-making. For this reason, having a low threshold to engage in shared decision-making (and documenting these discussions) may also serve to mitigate legal risks.

In the fourth case, a patient had symptoms including unilateral facial weakness and ear pain that could be consistent with Bell palsy. However, she did not respond to treatment over several months and received no instructions for follow-up. While some patients with Bell palsy will have permanent deficits, the majority will recover full function, and those who do not recover warrant additional evaluation. Failing to appreciate or communicate these follow-up parameters to patients at discharge can put them at risk, as occurred when this patient’s undiagnosed parotid gland cancer metastasized. While this additional evaluation is generally beyond the scope of the ED, discussing that such an evaluation may be necessary *is* the responsibility of the diagnosing physician. Documenting this discussion may help thwart any later allegations that such a conversation did not occur, and when this documentation is provided in the discharge instructions, it can help ensure patients have an avenue to revisit and better understand those recommendations, too.

## CONCLUSION

Bell palsy is the leading cause of unilateral facial paralysis, often presenting with a variety of symptoms consistent with dysfunction of the seventh cranial nerve. Patients with facial paralysis frequently seek emergency care due to the sudden onset and distressing nature of the condition. Given that more serious and potentially treatable conditions can also present with facial weakness, it is essential to conduct a comprehensive history and physical examination to differentiate Bell palsy from life-threatening causes, including acute stroke. From a legal perspective, fully documenting the care provided, including any shared decision-making and complete discharge instructions, may help mitigate downstream risks.
